# Restorative dentistry clinical decision-making for hypodontia: complex cases

**DOI:** 10.1038/s41415-023-6324-5

**Published:** 2023-10-13

**Authors:** Adrian Pace-Balzan, Andreas Chatzipantelis, Katharine J. Dunn, Garima Charan, Martin P. Ashley

**Affiliations:** 41415387399001 Restorative Dentistry, Edinburgh Dental Institute, UK; 41415387399002 Restorative Dentistry, Eastman Dental Hospital, London, UK; 41415387399003 Restorative Dentistry, University Dental Hospital of Manchester, UK

## Abstract

Hypodontia is a relatively common condition and patients will be seen by both general dental practitioners and specialist dental colleagues. Although hypodontia can be described as mild, moderate and severe, this does not directly correlate with the complexity of treatment required to provide an acceptable outcome. In addition, the complexity of treatment provided by one colleague in the multidisciplinary team may not be the same as for other colleagues.

When treatment planning and delivering dental care for these patients, especially those with severe hypodontia, it is useful to recognise the factors that make their care complex and also to follow principles for multidisciplinary treatment planning.

## Key points

- To understand the concept of complexity in patient care and the challenges of managing patients with severe hypodontia.
- To consider the factors related to complexity for hypodontia patients.
- To understand the main decision-making principles when managing complex hypodontia.

## Introduction

The overall prevalence of hypodontia, excluding third molars, is around 6.4% worldwide. The prevalence of mild (1-2 missing teeth), moderate (3-5 missing teeth), and severe hypodontia (6+ missing teeth) is 81.6%, 14.3%, and 3.1%, respectively.^1^

Hypodontia is frequently associated with delayed eruption patterns and alterations in tooth position and crown and root morphology. These dental variations are often accompanied by growth disturbances and discrepancies in the maxillofacial region, which may have a negative impact on occlusion and facial appearance. Patients with more severe hypodontia showed tendencies to a Class III skeletal relationship and a reduced maxillary-mandibular plane angle.^2^ Significant Class II and Class III incisal relationships are also more common with severe hypodontia.

The management of patients with hypodontia can therefore be inherently complex, often necessitating multidisciplinary care, as originally recommended by Hobkirk and colleagues.^3^

## The definition and assessment of complexity

Complexity can be defined as 'the state of having many parts and being difficult to understand or find an answer to'. In health care, several methods have been used to assess patient complexity, in recognition of the burden that complex patient care places on patients, health care systems and society.

In restorative dentistry, one validated tool to assess complexity is the Restorative Dentistry Index of Treatment Need (RDITN).^4^ Treatment need is subdivided into three domains of which complexity of treatment is one. This domain is assessed by clinicians and is subdivided into three skill levels (graduate, experienced and specialist) and at speciality level (periodontics, endodontics and prosthodontics). Assessment of complexity of care also includes patient-specific biomedical and psychological factors (modifying factors) leading to a summative score: the restorative dentistry complexity code. However, health literacy, socioeconomic, cultural, environmental and behavioural factors also contribute to patient complexity^5^ and these are not assessed by the RDITN. This index has not been widely used in clinical practice.

Ahmed and colleagues have considered and illustrated the assessment of case complexity, based on current national guidelines, when secondary care referrals for restorative dentistry are made.^6^ NHS England published the *Clinical standards for restorative dentistry* to support the commissioning of clinical services. This defines the procedures and modifying patient factors that describe the complexity of a case. Levels 1, 2 and 3 care descriptors outline the complexity of the clinical care required, including planning, technical/operative procedures and any modifying patient factors. They reflect the competence of the clinician and the setting required to deliver care of that level of complexity and may change, depending upon one or more patient factors.^7^

Unsurprisingly, assessment of the complexity of a 'hypodontia case' can be challenging, with numerous clinical and personal factors to consider. Complexity of treatment may also be different for the various specialties involved in the care of the hypodontia patient and the delivery of complex care may lie unevenly between the different specialties.

Although hypodontia can be described as mild, moderate and severe, this does not directly correlate with the complexity of treatment required to provide an acceptable outcome.

**Fig. 1:**
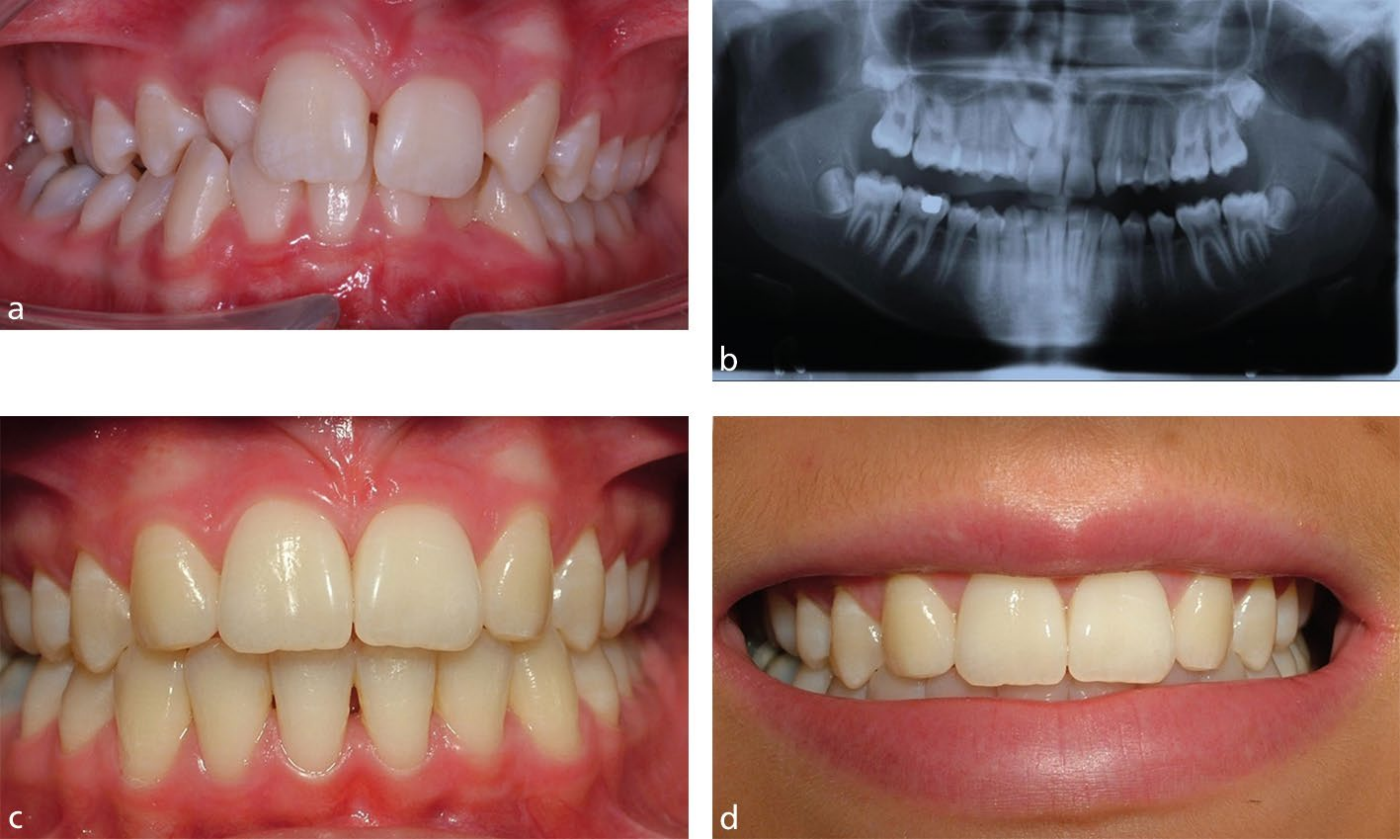
a, b, c, d) Teenage patient. Crowded dentition, impacted upper right canine tooth and mild hypodontia with missing upper lateral incisors

For example, a patient with a crowded dentition, impacted upper right canine tooth and mild hypodontia with missing upper lateral incisors may require surgical management and lengthy and complex orthodontic treatment with little, if any, requirement for specialist restorative dentistry treatment (Fig. 1).

**Fig. 2:**
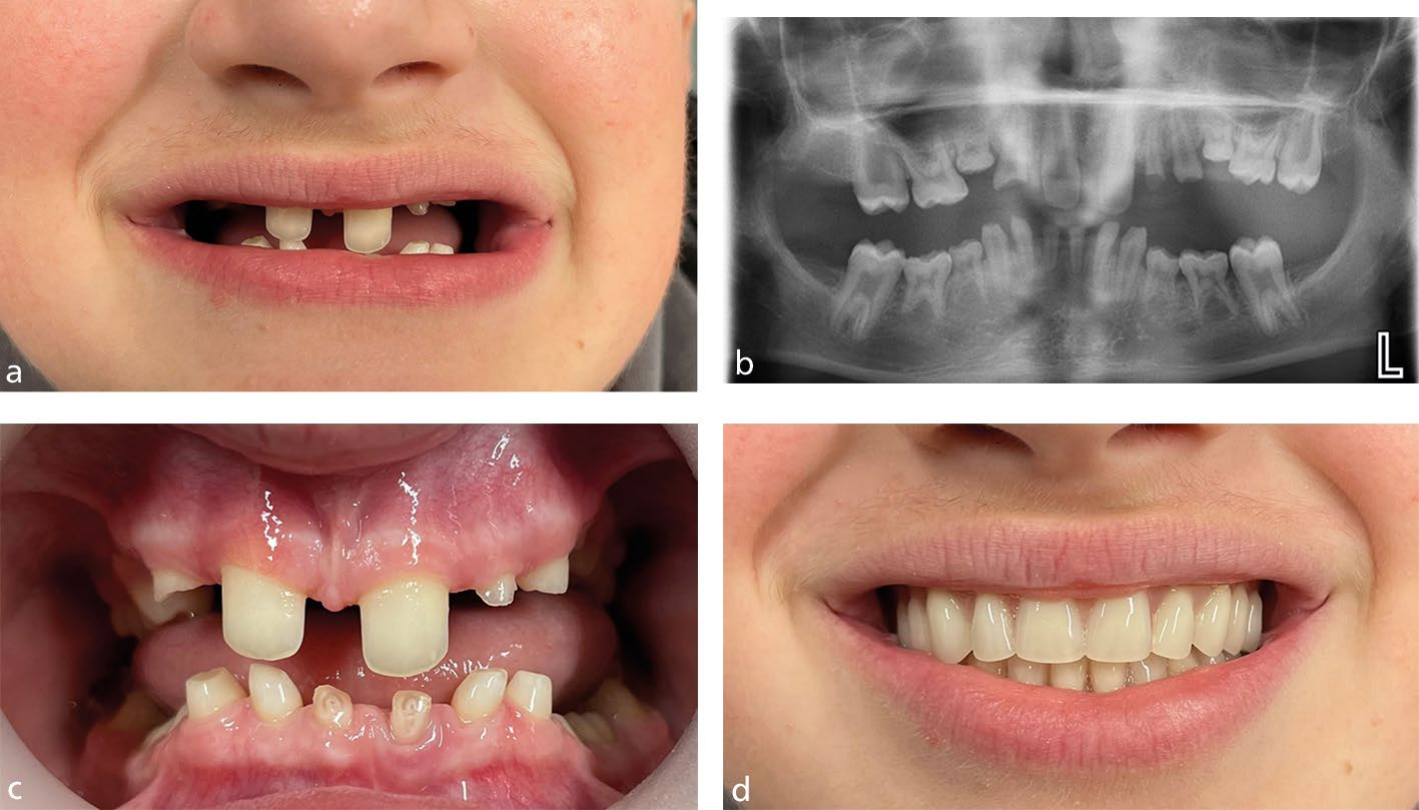
a, b, c, d) Teenage patient. Severe hypodontia: only six permanent teeth have developed and has several retained deciduous teeth. This patient may not benefit at all from orthodontic treatment and initially, only require specialist restorative dentistry treatment in the form of removable complete overdentures. These meet the patient's functional and aesthetic requirements and are an important stage in what may be a lifelong requirement for more complex dental care

However, a patient with severe hypodontia, who has developed only six permanent teeth and has several retained deciduous teeth, may not benefit at all from orthodontic treatment and initially only require specialist restorative dentistry treatment in the form of removable complete overdentures (Fig. 2).

**Fig. 3:**
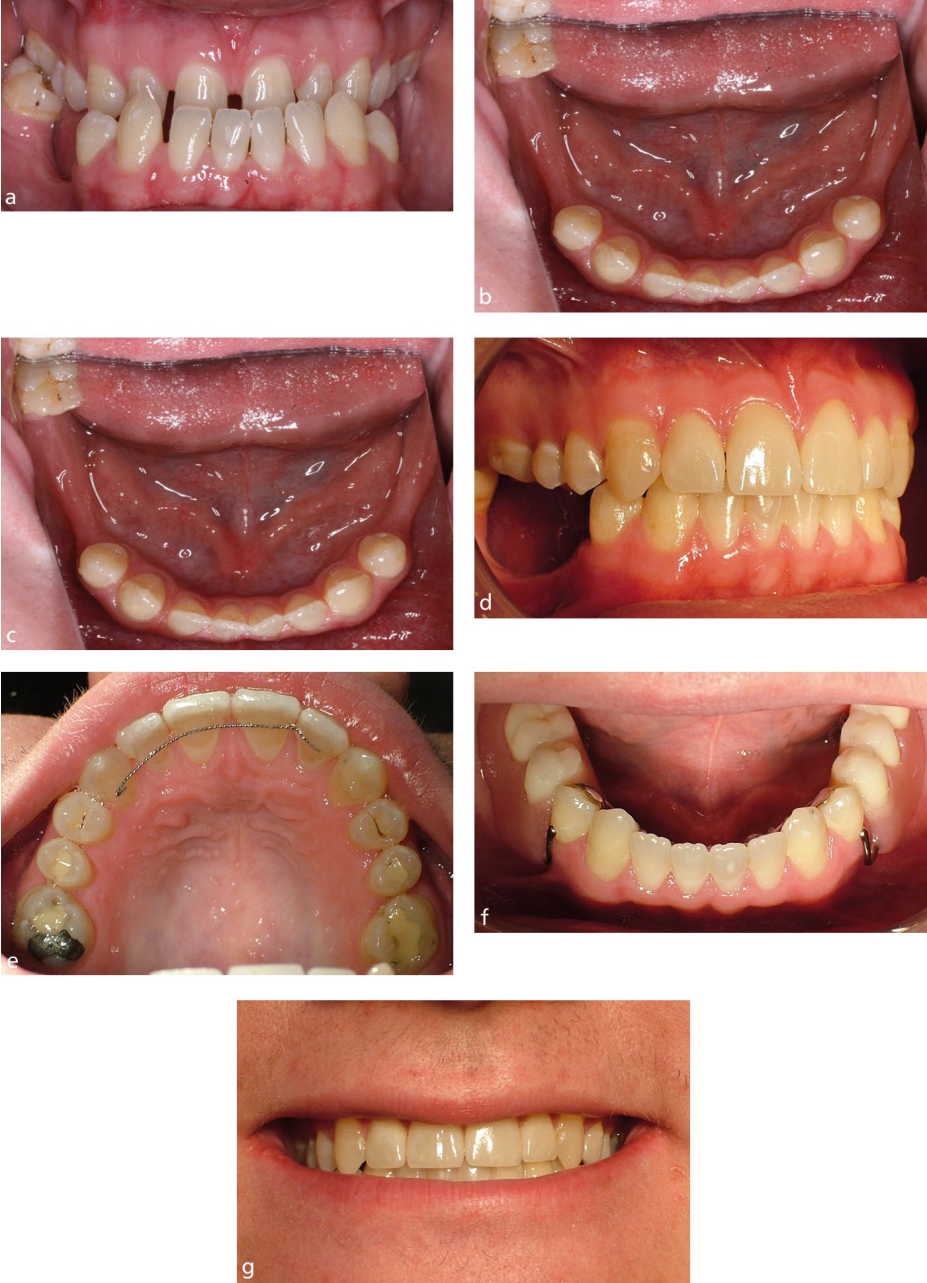
a, b, c) Young adult patient. Severe hypodontia of posterior teeth, spaced and relatively narrow upper anterior teeth, with a Class III skeletal relationship. d, e, f, g) This patient benefited from orthodontic and orthognathic surgical treatment, followed by veneer restoration of their upper anterior teeth and a removable lower partial denture to replace the missing lower posterior teeth. This required 37 appointments over a period of four years and five months

Alongside these, some complex hypodontia patients benefit from a truly multidisciplinary management approach to address their functional and aesthetic requirements (Fig. 3).

## Factors related to the challenge of managing a patient with complex hypodontia

### Skeletal and occlusal relationships

There is an increased tendency for patients with severe hypodontia to present with either Class II or Class III incisal and skeletal relationships, requiring complex orthodontic and restorative dentistry management and potentially orthognathic surgical care.

### Reduced alveolar development in sites of missing permanent teeth

This may prevent orthodontic movement of a tooth into a more ideal position. From a surgical perspective, it may not be possible to provide predictable alveolar augmentation for dental implant placement, required for a reliable and acceptable treatment outcome. Reduced alveolar development can also negatively affect the gingival level against which a bridge pontic is placed, requiring an unacceptable higher cervical margin. Overall, these factors may necessitate a removable, rather than a fixed prosthesis. This can reduce the complexity of treatment but may not meet many patients' desires for a fixed restoration.

### Retained deciduous teeth at presentation

This can mask the likely future impact on aesthetics and function for a patient with severe hypodontia. Ankylosis and infraocclusion of deciduous molar teeth are common. The rate of root resorption of deciduous teeth with no permanent successor is variable and these teeth can either exfoliate as expected and require replacement or be retained late into adult life and cause aesthetic, functional and treatment planning difficulties for the patient and the clinical team.

### Delayed eruption and impaction of permanent teeth

This will require additional surgical intervention, often before orthodontic treatment can commence. Due to the patient's young age and the surgical complexity, such treatment usually involves a general anaesthetic. At times, hospital services may give such treatment a lower priority than other more urgent surgical procedures, therefore significantly lengthening the overall treatment process, and potentially affecting what treatment options can be offered.

An impacted permanent tooth can cause resorption of other, adjacent permanent teeth.

Surgical removal of an impacted tooth can lead to significant, post-extraction remodelling of the alveolar ridge, affecting the achievement of an acceptable aesthetic outcome.

### Disproportionate and asymmetric spacing of natural teeth

This can cause increased difficulties for orthodontic tooth movement required for ideal three-dimensional space creation to allow preferred replacement of missing teeth.

### Shape and size of teeth

Microdontia is commonly associated with hypodontia. Crown and root morphological differences are also present, including small, conical and tapered crown forms and root abnormalities. Achieving a reliable bond between orthodontic brackets and diminutive teeth can be difficult, preventing the ideal three-dimensional positioning of the teeth.

Providing interim and definitive restoration of diminutive teeth is often useful and desirable. However, achieving an acceptable outcome is challenging, given the narrow cervical emergence profile, reduced surface area for bonding, poor retention and resistance form and unfavourable crown-root ratios.^8^

### Rotation and angulation of teeth and unfavourable three-dimensional positioning of teeth

**Fig. 4:**
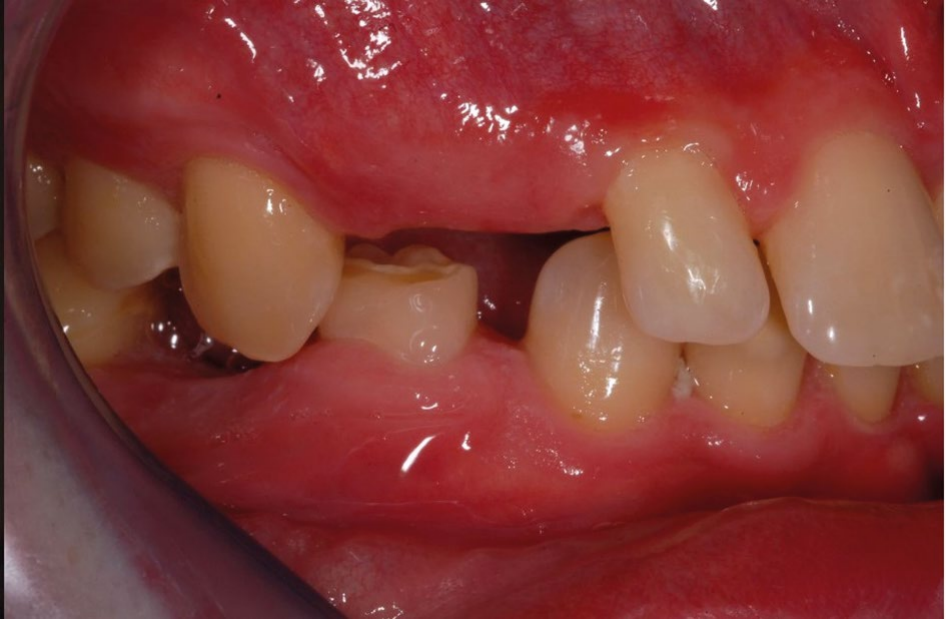
Young adult patient with severe hypodontia. Stopped wearing retainers and as a result, the relapse led to loss of sufficient interocclusal space required for tooth replacement. A further course of orthodontic treatment is required

The unfavourable eruption of teeth in a patient with severe hypodontia can complicate orthodontic and restorative dentistry treatment. Relapse following orthodontic treatment, particularly with inadequate retainer wear, can result in unacceptable tooth positions within the same arch and unfavourable inter-occlusal relationships, preventing sufficient three-dimensional space for ideal restorations (Fig. 4).

**Fig. 5:**
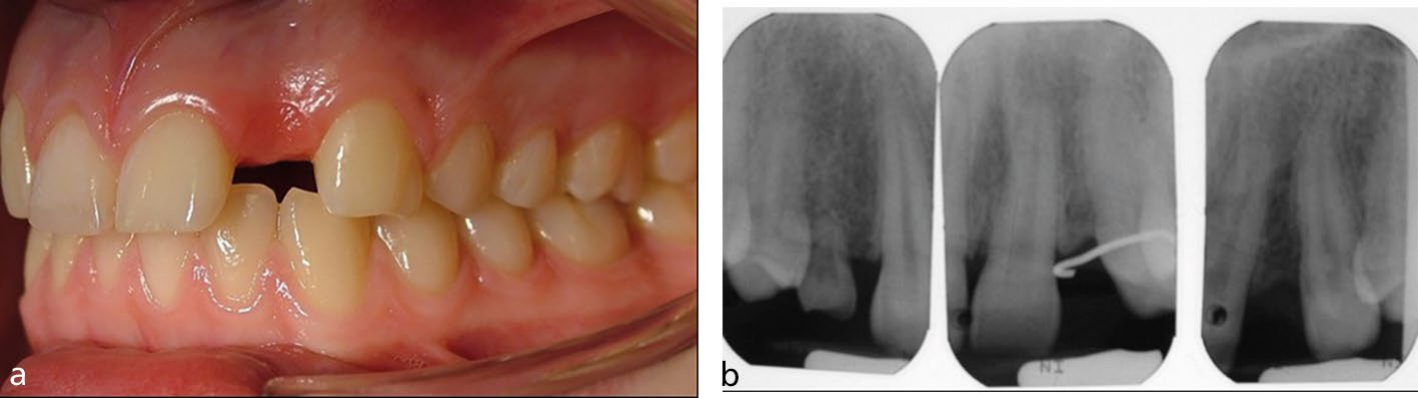
a, b) Young adult patient with missing upper lateral incisor teeth, who has completed a course of orthodontic treatment without any restorative dentistry involvement in either the treatment planning process or assessment before debond. The patient was referred, anticipating dental implant treatment to replace the two missing teeth. Despite the apparent coronal space, unfortunately, the upper left lateral incisor space is unsuitable for a dental implant, as the adjacent roots converge

Converging roots of teeth adjacent to a space, despite the apparent correct orthodontic outcome when only considered from the coronal tooth positions, may prevent implant placement (Fig. 5).

### Potential for loss of tooth vitality and restorability

There are increased risks to tooth vitality of deciduous molar teeth if these are reshaped to facilitate the orthodontic creation of ideal premolar spaces. There are also risks to tooth vitality of any permanent teeth if they require preparation as abutments for conventional indirect restorations.

### Progressive deterioration and failure of an extensively restored dentition

**Fig. 6:**
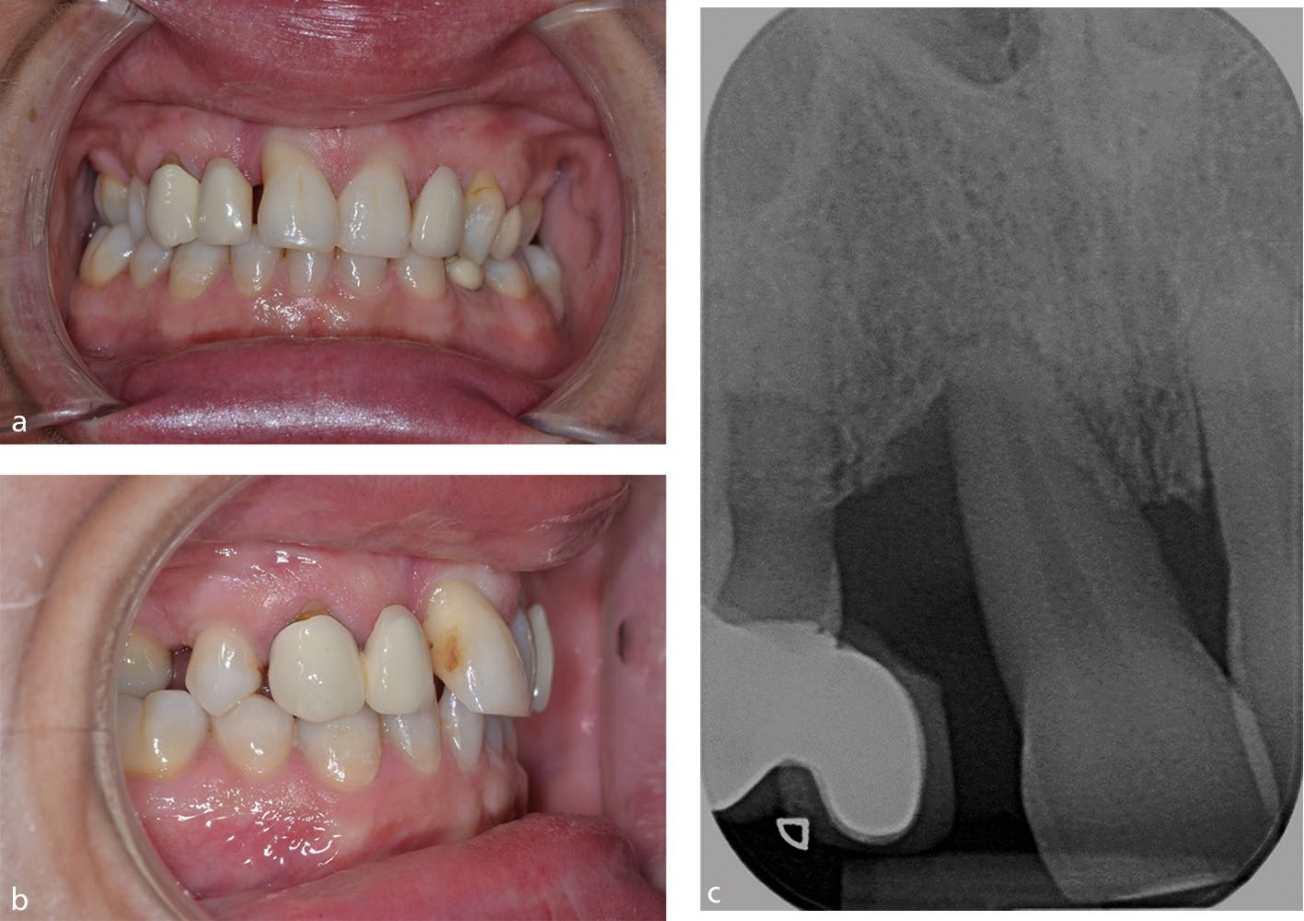
a, b, c) Adult patient previously treated for mild hypodontia. Loss of retention and localised Stage IV Grade C periodontitis resulted in loss of the central incisor tooth

When an adult patient returns seeking further treatment and the natural dentition has previously been extensively restored, there is an increased risk of caries and endodontic disease affecting the suitability of these teeth for a subsequent course of treatment. There is also the potential for difficulty in meeting the patient's expectations of further long-term fixed restorations, especially if their current abutment teeth have become unrestorable (Fig. 6).

## General considerations in the management of patients with complex hypodontia

As patients with severe hypodontia will usually require a prolonged and complex course of treatment provided by a number of clinical teams, the patients and their families face distinct challenges. They will usually require a large number of appointments with the specialist clinicians, often at significant distances from their home, leading to increased time away from school and work and the financial costs associated with these.^9^ For a family with more than one child affected by severe hypodontia, this can be an even greater burden.

Members of the clinical teams with shared responsibility for the patient can change during the prolonged treatment period and therefore, regular communication is a necessity, co-ordinating care between the multiple specialties.^10^ All members of the multidisciplinary team must agree on a treatment plan and assign milestones and treatment aspects to relevant team members.^11^ Treatment must also address the age-dependent needs of the patient within the context of primary and preventive care, delivered by the general dental practitioner, who should be included in all correspondence related to the patient's treatment.

**Fig. 7:**
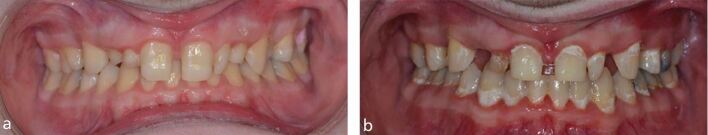
a, b) Teenage patient with hypodontia. Evidence of generalised plaque-induced gingivitis before the start of orthodontic treatment that deteriorated significantly during treatment and leading to early debond. Restoration is prevented due to inadequate oral health and would have been complicated by poor tooth positions due to early debond

Most patients with severe hypodontia will commence their treatment within early teenage years and with the full support of their families. As the treatment may be prolonged, the patient transitions to a time when they are old enough to become more involved with the clinical decision-making process. Equally, the patient assumes increasing responsibility for complying with treatment and, for example, maintaining a healthy diet and adequate daily dental hygiene. If they do not achieve this, treatment may need to be either suspended or even discontinued (Fig. 7).

## The goals of managing patients with complex hypodontia

From a restorative dentistry perspective, the goals of treatment are to provide acceptable dentofacial appearance and oral function, with suitable restoration longevity, to improve overall patient wellbeing and quality of life. Ideal aesthetic and functional treatment outcomes are necessary against which to plan treatment, and this can be modified according to what is clinically possible, to the patient's concerns and wishes, and their commitment to the treatment process. The patient must also be made aware of the implications of maintenance and replacement of restorations throughout their lifetime.

Early involvement of the restorative dentist in multidisciplinary planning is essential to identify issues in relation to the planned long-term aesthetic and functional outcomes. The responsibility is to plan and where necessary, gain agreement with the orthodontist and oral surgeon on the final skeletal relationship, the sites and dimensions of edentulous spaces and the desired functional occlusion.^12^ The restorative dentistry specialist will anticipate potential aesthetic and functional difficulties and precisely guide the orthodontist, in terms of final tooth positions, to have several tooth replacement options to consider with the patient.

## Treatment planning principles for patients with complex hypodontia

Regardless of the number of teeth that fail to develop, all clinical presentations for hypodontia may present complexity. When planning treatment, it can be useful to follow certain principles that can be adapted to each individual patient. For severe hypodontia, these principles may be subtly different from either mild or moderate hypodontia.

It is likely that most patients would commence the treatment planning process favouring a fixed definitive treatment outcome, using bridges or dental implant supported restorations, rather than a treatment outcome, based either partly or entirely on removable dentures. However, given the increased complexity, treatment duration, number of appointments, requirement for surgery, greater clinical risk, increased financial costs of treatment and maintenance burden to achieve an entirely fixed treatment solution, it is appropriate that removable prosthetic treatment options are also considered. If this does not provide an acceptable long-term outcome for the patient, it is then possible to explore the fixed restoration options.

### Complete or almost complete agenesis of permanent teeth

This is a very rare occurrence and generally, affected patients present during childhood and can be successfully managed with removable complete dentures or overdentures. As these patients have learned to eat and communicate without teeth, providing a denture is usually initially required to improve their dental appearance. Therefore, patients often prefer to just use the upper denture, even though both may be provided. The early adaptation period can be challenging for the young patient, especially if learning to use a denture while attending school with their peers. The clinical team, sensitive to these issues, will consider providing the denture to coincide with the start of a school holiday period, so the patient can adapt more readily, away from the potentially unsupportive school environment.

**Fig. 8:**
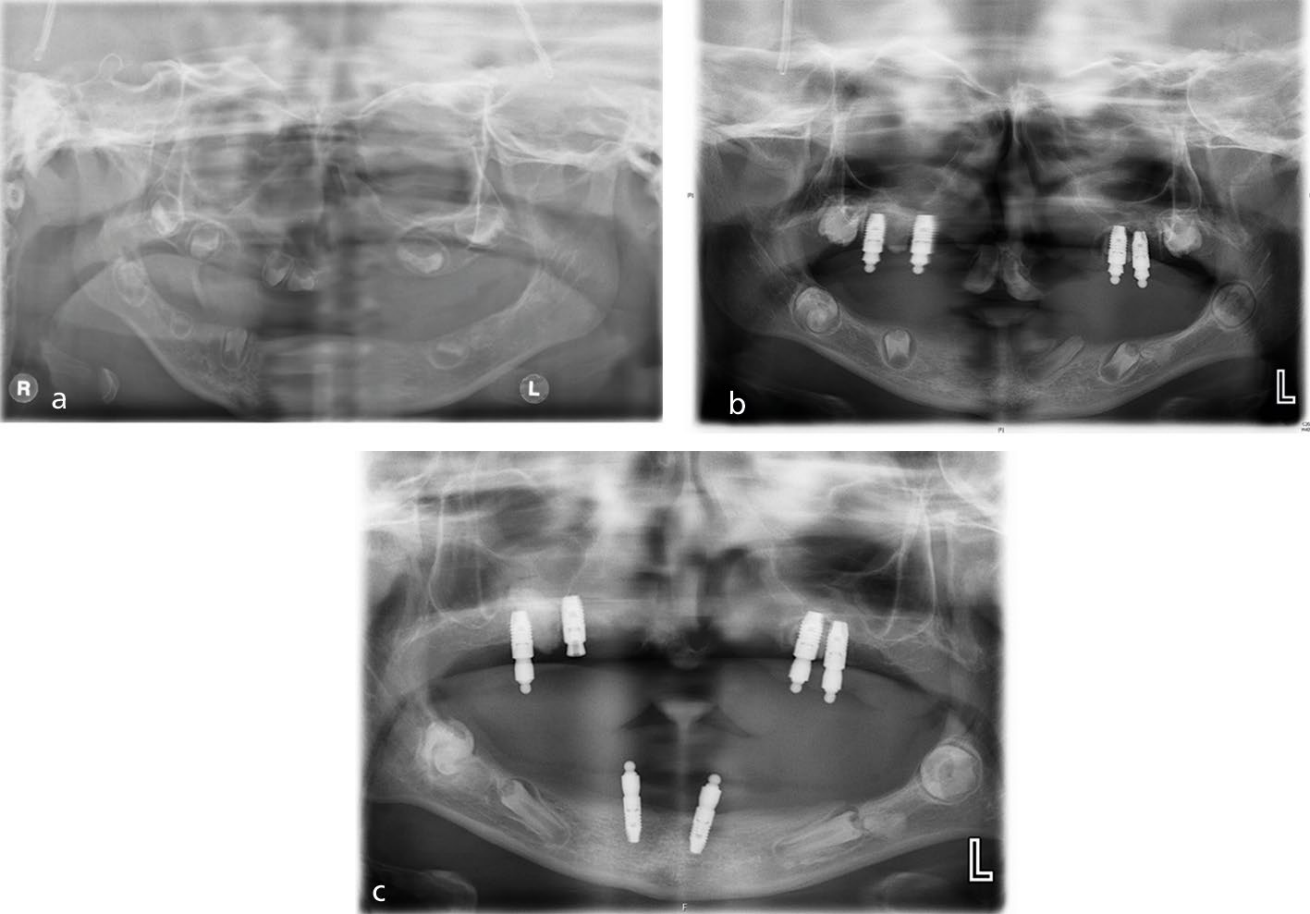
a, b, c) Panoramic radiographs taken in 2012, 2015 and 2018, of a child with ectodermal dysplasia and bilateral cleft lip and palate, demonstrating the progressive use of dental implants in the developing patient. Reproduced with permission from L. Clarke *et al.*, 'Britain's youngest implant patients? - A Case Series of implant treatment in children with ectodermal dysplasia', *Oral Surgery*, 2020, John Wiley & Sons^13^

**Fig. 9:**
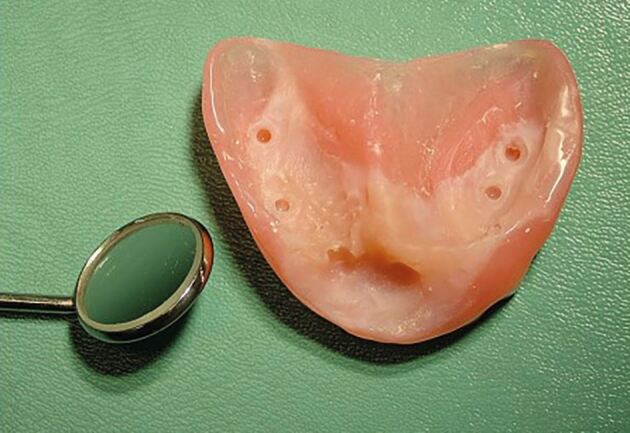
Image of the fitting surface of the first denture provided for the same child from Figure 8. The small size of the denture can be seen, relative to a dental mirror

Some patients, particularly if syndromal or affected by a cleft palate, may not have sufficient denture-bearing anatomy to wear a denture and therefore can be considered for early dental implant treatment, used to assist in the retention of the denture^13^ (Fig. 8, Fig. 9).

Ongoing oro-facial development requires denture(s) to be replaced on a regular basis through childhood and additional dental implant surgery can be provided as the facial skeleton develops.

Denture(s) can also be useful in the diagnostic and planning processes for future treatment stages, particularly if involving fixed restorations.

For a patient with severe hypodontia, the teeth most likely to develop are the upper central incisors, upper and lower canines and first molar teeth. Patients with these few teeth may be managed with removable partial or complete overdentures but often benefit from some initial orthodontic treatment. The aim of this is to address a frequent patient complaint of a gap between their upper teeth. By aligning the upper central incisor teeth together and coincident with the facial midline, other visible gaps can be filled with the partial denture or overdenture. This approach improves the young patient's self-confidence related to their dental appearance, introduces them to the benefits of dental treatment, and assists with the longer-term tooth positioning and treatment planning processes. However, some young patients who wear dentures may express some level of body image dissatisfaction and psychological morbidity.^14^

### Severe hypodontia with failure of development of many permanent teeth

These patients, who are missing more than six permanent teeth, can present some of the greater treatment challenges, in terms of tooth positioning, tooth shape and options for replacing the missing teeth. Some principles to guide the treatment planning process are:

1. Create symmetry on each side of the facial and dental midline
2. Orthodontically either reduce or close spaces, to reduce the short- and long-term treatment burden of having prosthetic replacement for the missing teeth
3. Where spaces are necessary, ideal three-dimensional positioning of the adjacent teeth and consideration of inter-arch occlusal relationships maintain the options for prosthetic replacement of the missing teeth with dentures, bridges or dental implants. A combination of these restoration options may be required to achieve an acceptable aesthetic and functional outcome (Fig. 3)
4. Where possible, replace single missing teeth with resin-retained bridges. Treatment can be completed in as little as two, non-invasive appointments and this approach does not preclude the longer-term use of dental implants, if required. With the standardisation of resin-bonded bridge design and clinical techniques, this is a valid, minimally invasive, aesthetic, reversible and predictable treatment solution, when employed in carefully selected clinical situations.^15^^,^^16^^,^^17^ Although the consensus is that cantilever resin-bonded bridges provide a longer-term and more predictable solution than fixed-fixed resin-bonded bridges, the latter design may be a consideration in view of the potential orthodontic relapse and loss of interradicular space that may compromise the placement of dental implants at a later stage in the patient's treatment journey. Longer-span, fixed-fixed design resin-bonded bridges can also be successful in the replacement of all four lower incisor teeth if the adjacent canine shape and the anterior occlusion are favourable. The lack of alveolar development in this area may make predictable dental implant treatment impossible
5. For other spaces requiring more than a single tooth replacement, a removable partial denture will replace both the missing teeth and soft tissues and can provide an acceptable short- or long-term treatment solution
6. For most, but not all spaces, replacement of the missing teeth with dental implant restorations can be considered, usually after conventional treatment options, such as dentures and bridges, have been attempted. However, anatomical constraints, such as the lack of alveolar development, convergence of adjacent teeth and proximity of the inferior dental nerve, may complicate or even prevent this surgical approach. Patients should be made aware of the increased complexity and duration of dental implant treatment and the requirement for maintenance and retreatment. This option also has relatively significant financial costs and while for suitable cases these may be initially met by, for example, local NHS commissioning arrangements in England, this cannot be relied upon for further courses of treatment, potentially leaving the patient to manage this financial burden.^18^

## Recent advances in the management of patients with complex hypodontia

In recent years, advances in three-dimensional imaging and computer-aided design/computer-aided manufacturing technology have resulted in the beginnings of a potential paradigm shift in patient care. Digital technology and workflows offer improved possibilities in the diagnosis, treatment planning and effective delivery of surgery and dental restorations. These can be used to simulate and present the proposed orthodontic, orthognathic and dental outcomes to the patient and family. By means of data from computed tomography or cone beam computed tomography scans and facial and oral photographs, a virtual face, with craniofacial and dentoalveolar bone and coloured soft tissues, can be created using 3D imaging software and the use of bony and soft tissue points plotted on the virtual face.^19^ These images are superimposed and manipulated such that digital Kesling set-ups, diagnostic digital 'wax-ups', virtual osteogenic distraction and orthognathic surgery and guided implant planning and placement are planned and predictably delivered.

## Conclusion

All clinicians will meet patients who have complex clinical needs and a proportion of these patients will be affected by hypodontia. Their condition will often start to become evident in late childhood or early teenage years, but patients may present later in life with an extensively restored dentition that is beginning to fail. It is important that general dental practitioners are confident to assess these patients, make a referral to a specialist colleague if appropriate and contribute to long-term patient care as part of the multidisciplinary team. Specialist colleagues will collaborate closely together, especially in the treatment planning process, to ensure the patient's complex condition is managed appropriately and an acceptable outcome is achieved.
